# Phospholipid Phosphatase 4 as a Driver of Malignant Glioma and Pancreatic Adenocarcinoma

**DOI:** 10.3389/fonc.2021.790676

**Published:** 2021-11-30

**Authors:** Wenxiu Tian, Ping Wang, Zhimei Wang, Huimin Qi, Junhong Dong, Hongmei Wang

**Affiliations:** ^1^ School of Basic Medicine, Weifang Medical University, Weifang, China; ^2^ Center of Translational Medicine, Zibo Central Hospital, Zibo, China; ^3^ Jiangsu Province Hi-Tech Key Laboratory for Biomedical Research, and School of Chemistry and Chemical Engineering, Southeast University, Nanjing, China; ^4^ Department of Pharmaceutical Sciences, Binzhou Medical University, Yantai, China

**Keywords:** phospholipid phosphatase 4, proliferation, tumorigenesis, glioma, pancreatic adenocarcinoma

## Abstract

Glioma and pancreatic cancer are tumors with a high degree of malignancy, morbidity, and mortality. The present study explored possible molecular mechanisms and potential diagnostic and prognostic biomarker-PLPP4 of glioma and PAAD. PLPP4 is differentially elevated in glioma and PAAD tissues. Statistical analysis from TCGA demonstrated that high expression of PLPP4 significantly and positively correlated with clinicopathological features, including pathological grade and poor overall survival in glioma and PAAD patients. Following this, the methylation levels of PLPP4 also affected overall survival in clinical tissue samples. Silencing PLPP4 inhibited proliferation, invasion, and migration in LN229 cells and PANC-1 cells. Moreover, the combination of multiple proteins for the prognosis prediction of glioma and PAAD was evaluated. These results were conducted to elaborate on the potential roles of the biomarker-PLPP4 in clonability and invasion of glioma and PAAD cells.

## Introduction

Invasion and metastasis are the basic characteristics and essential markers of malignant tumors, the leading cause of death in cancer patients. Patients with glioma and pancreatic adenocarcinoma (PAAD) have relatively shorter survival than patients with other malignancies. Malignant gliomas can broadly be considered those tumors as glioblastoma multiforme (GBM) and brain lower grade glioma (LGG) and remain among the most treatment-resistant cancers. Chemotherapy or radiotherapy can only be used as adjuvant therapy in treating malignant gliomas, and operative treatment cannot safely remove the entire tumor from the brain due to their locally invasive behavior. These treatments are still inevitably fatal (1). Therefore, the early prevention of malignant gliomas is crucial. Studies have found frequent mutations in chromatin modifiers, often defining specific glioma subtypes, and this epigenome reprogramming may be a fundamental driver of gliomas (2, 3). However, there are still few biomarkers for glioma prevention.

Another malignancy tumor with a relatively short survival is PAAD. Pancreatic neoplasms include cancers from the endocrine or exocrine components of the pancreas, of which pancreatic adenocarcinoma from the exocrine pancreas is the most common and most aggressive cancer affecting human health. The rates of pancreatic cancer are on the rise, having more than doubled worldwide in the past 30 years (4). PAAD is a common malignant tumor with a poor overall prognosis. The prognosis has been improved in patients with feasible resection and adjuvant therapy. According to the latest data, the 5-year overall survival rate of PAAD has increased slightly but remains <10% (5). The rare representative symptoms and diagnosis of PAAD are considered the leading cause of mortality and morbidity (6, 7). Surgical resection is the only possible treatment for patients with localized PAAD, but no more than 20% of patients are eligible for initial resection. Chemotherapy is the preferred treatment for patients with advanced PAAD, but chemotherapy has a wide range of side effects and is unsuitable for patients with low-performance status. With these shortcomings, other substitutive therapies are highlighted for early metastasis or recurrence. Therefore, early detection of PAAD is crucial to provide patients with the best treatment. In addition, a comprehensive analysis of accurate prognostic biomarkers is needed to help guide patient treatment. It is essential to identify candidate biomarkers for the diagnosis and prognosis of PAAD. In addition, an in-depth understanding of potential biomarkers and the therapeutic targets of PAAD will facilitate the development of a novel therapeutic strategy.

Lipid phosphate phosphatases (LPPs), also known as phospholipid phosphatases (PLPPS), belong to the complete membrane glycoprotein superfamily, with six transmembrane domains and three highly conserved domains. The conserved domains are paralleled to the transmembrane domain and form the active site of phosphatase (8, 9). In addition, there is increasing evidence that abnormal expression of LPPs is associated with the development and progression of cancer. For example, lower expression of lipid phosphate phosphatase-1 and - 3 (PLPP1 and PLPP3) was significantly associated with worse OS (overall survival) in lung adenocarcinoma (LUAD) patients. Expression of PLPP3 was positively correlated with tumor-infiltrating immune cells in non-small-cell lung cancer patients (10).

Meanwhile, PLPP3 was downregulated in oral squamous cell carcinoma (OSCC) patients, and PLPP3 expression negatively correlated with TNM stage and tumor volume (11). Moreover, PLPP5 was found in several cancers, including breast cancer, pancreatic adenocarcinoma, and lung carcinoma (12). PLPP4 is observed in lung carcinoma tissues and cells and positively correlates with advanced clinicopathological features and poor prognosis in lung carcinoma patients (13). Thus, these studies indicated that different LPPs exert oncogenic or tumor-suppressive functions depending on the tumor types.

Although the expression and functions of LPPs have been reported in some studies, the general characterizes of LPPs as targets and biomarkers in glioma and PAAD are largely unclear. A synthetical analysis of the roles of LPPs in glioma and PAAD has become urgent at present. In this study, by analyzing the expression levels of LPP family proteins in tumor RNA expression profile datasets from The Cancer Genome Atlas (TCGA), we found that PLPP4 is dramatically elevated compared with other LPPs in the glioma and PAAD tissues. Our findings indicate that PLPP4 has the potential values as clinical markers and immunotherapeutic targets in the glioma and PAAD based on multiple large bioinformatics databases, thus providing clinicians with additional information to help them choose more appropriate drugs and more accurately assess the prognosis of the glioma and patients.

## Materials and Methods

### UALCAN

UALCAN (http://ualcan.path.uab.edu/analysis.html), a comprehensive and interactive web resource, provides easy access to publicly available cancer OMICS data (TCGA and MET500) (14). Our study obtained LPPs level in the “Expression” links using the “TCGA analysis” module and the glioma and dataset.

### GEPIA

GEPIA (http://gepia.cancer-pku.cn/index.html) is a developed interactive web server for analyzing the RNA sequencing expression data of tumors and normal samples from the TCGA and the GTEx project (15). In this study, we performed the pathological stage analysis and multiple gene comparison analysis of LPPs. And the correlation between PLPP4 and disease-free survival (DFS) was also calculated.

### Kaplan–Meier Plotter

Kaplan–Meier Plotter (https://kmplot.com/analysis/) is a useful prognostic biomarker assessment tool that explored the effect of 54k genes on survival in 21 cancer types using the databases from GEO, EGA, and TCGA to analyze the prognostic value of PLPP4 in PAAD overall survival.

### MethSurv

MethSurv (https://biit.cs.ut.ee/methsurv/), a web tool for survival analysis based on CpG methylation patterns, was applied to explore the prognostic value of single CpG methylation of PLPP4 in glioma and PAAD patients (16).

### SurvivalMeth

SurvivalMeth (http://bio-bigdata.hrbmu.edu.cn/survivalmeth/) was used to analyze the DNA methylation of PLPP4 signature on glioma and PAAD prognosis (17).

### EMBL-EBI

EMBL-EBI (https://elixir-europe.org/about-us/who-we-are/nodes/embl-ebi), a flexible pipeline for single cell RNA-seq analysis that integrates many existing tools for filtering and mapping reads, quantifying expression, clustering, finding marker genes and variable genes (18).

### Timer

Timer web server (https://cistrome.shinyapps.io/timer/) is a comprehensive resource for systematical analysis of the infiltration of different immune cells and their clinical impact across diverse cancer types (19, 20).

### Cell Culture

The human glioma cell lines LN229, U251, U87MG, SHG-44, T98G, and PAAD cell lines AsPC-1, BxPC-3, PANC-1, HPAF-11, Hs766T were obtained from ATCC (The American Type Culture Collection). All cells were cultured in DMEM medium (Life Technologies, Carlsbad, CA, US) supplemented with penicillin (100 U/ml), streptomycin (100 mg/ml), and 10% fetal bovine serum (FBS, Life Technologies) and were grown under a humidified atmosphere of 5% CO_2_ at 37°C.

### Construction of Short Hairpin RNA Targeting PLPP4

According to the reference, based on the PLPP4 cDNA sequence in GenBank (NM_001030059.2). The interference sequence of target protein was shRNA-PLPP4: 5’-GGAGTGATGAACTCGGAAAATG-3’, and the negative control sequence was 5’-ACTACCGTTGTTATAGGTG-3’ (13); two DNA single strands expressing shRNA-PLPP4 complement connected to pGV248⁃SC1⁃PLPP4 (purchased from Shanghai Jikai Biotechnology Co., LTD.), and constructed lentivirus recombinant vector pGV248⁃SC1⁃PLPP4. HEK293T cells are then used for packaging the virus. When the degree of LN229 and PANC-1 cells fusion reached 85%, 3x10^4^/mL cell suspension was prepared and inoculated on 6-well plates. When the cell density reached 30-40%, the shRNA-Con group and shRNA-PLPP4 group were transfected with negative control lentivirus and shRNA-PLPP4 lentivirus, respectively. Cells were collected when the degree of cell fusion was about 80%, and the interference effect was detected.

### Construction of Targeted PLPP4 Overexpression

PLPP4 gene was synthesized by chemical synthesis method and connected to pcDNA3.1. The PLPP4 gene (BC132787.1 CDS region) with KpnI/XhoI enzyme cutting site was synthesized by chemical synthesis method, and the primers were F: 5’-GGGGTACCATGCGGGAGCTGG-3’ and R: 5’-CCCTCGAGTCACAGATCCTCTTCAG-3’, and connected to pcDNA3.1 through KpnI/XhoI after enzyme digestion. After sequencing, the vector and overexpressed plasmid were transfected into glioma cells LN229 and pancreatic cancer PANC-1 cells by liposo2000 (Invitrogen, Carlsbad, CA, USA), respectively.

### RNA Extraction, Reverse Transcription, and Real-Time PCR

Total RNA from tissues or cells was extracted using the RNA Isolation Kit-miRNeasy Mini Kit (Qiagen, USA) according to the manufacturer’s instructions (21). According to the manufacture’s protocol, the messenger RNA (mRNA) was reverse transcribed from the total mRNA using the Revert Aid First Strand cDNA Synthesis Kit (Thermo, USA). Complementary DNA (cDNA) was amplified and quantified on a CFX96 system (BIORAD, USA) using iQ SYBR Green (BIO-RAD, USA). The upstream primers of PLPP4 were 5’-TTTGGATCCGTTCCAGAGAG-3’and the downstream primers were 5’-CAGGGGTGTGAGGAAAGAAA-3’. The upstream primer of β-actin was 5’ -CATGGGCCAGAAGgACTC-3’, and the downstream primer was 5’-AAGGTCTGGAGCCAGATC-3’. Amplification conditions: 95°C for 2 min, 95°C for 30s, 60°C for 30s, 72°C for 30s, 35 cycles. Relative fold expression was calculated using the comparative threshold cycle (2-ΔΔCt) method.

### Western Blotting

Total protein of LN229 cells and PANC-1 cells in each treatment group was extracted. The exact amount of protein was taken for SDS-PAGE electrophoresis, then transferred to NC membrane, containing 50 g/L skim milk powder sealed for 1h. Rabbit anti-PLPP4 antibody (1:1000, PA5-116155, Invitrogen) and mouse anti-β-actin (1:2000, 66009-1, Wuhan Sanying Biotechnology Co., LTD., China) were added and incubated at 4°C overnight. TBST was washed 3 times, 10 min each, and HRP labeled goat anti-rabbit IgG (1:4000), rabbit anti-mouse IgG (1:8000) were added, and incubated at room temperature for 1.5 h. TBST was washed 3 times, 10 min each, ECL substrate was colored, and the absorbance (A) value of the protein bands was scanned and analyzed.

### Cell Counting Kit-8 Analysis and Colony Formation Assay

For cell counting kit-8 analysis, cells (3 × 10^3^ were seeded into 96 well plates, and the specific staining process and methods were performed according to the previous study (22). Each group is provided with 3 parallel holes. The cell growth was observed at 0, 1, 2, 3, 4, and 5 days, and 10 μL CCK-8 was added to the culture for 4h. The A value of each well was detected by the absorbance microplate reader.

### Scratch Test

Con group (LN229 cells or PANC-1 cells), vector (pcDNA3.1), overexpression, shRNA-Con, shRNA-PLPP4 cells were digested with trypsin, and the counting cells were inoculated on 6-well plates. Scratch with pipette tip when cells fuse. The plate was rinsed twice with a fresh medium to remove adherent cells. Cell migration was observed and photographed at 0 and 24h. Cell migration at the wound edge was quantified and presented as the mean ± SD.

### Transwell Experiment

Take 100 μLMatrigel, and 300 μL precooled serum-free medium and mix well, add 50 μL of the above prepared matrix glue into Transwell™ well, and set at room temperature for 1h. The number of logarithmic growth cells in each group was adjusted to 2×10^5^ cells/ml, and 200 μL of the above cell suspension was added into the upper chamber of Transwell™. Complete medium 600 μL was added to the lower chamber of the culture plate and cultured for 24h. Fixed with 40 g/L paraformaldehyde for 15 min, stained with 2 g/L crystal violet for 20 min, counted five different fields at 100 times, and calculated the average value.

### Immunohistochemistry

The immunohistochemistry procedure and scoring of PLPP4 expression were performed as previously described (23). The glioma and PAAD tissue were cut into slices of 8um and placed in an oven at 65°C for 2 h. The sections were incubated in 3% hydrogen peroxide solution for 10 minutes and washed with PBS 3 times. The sections were placed in boiling EDTA repair solution for 20 min for antigen repair. The slices were washed with PBS 3 times, 5 minutes each, and sealed with 5% BSA for 20 minutes. The slides were incubated overnight at 4°C in a humidified chamber with anti-PLPP4 antibodies (Novus Biologicals: NBP2-14545) diluted 1:100 in PBS. Wash with PBS 3 times, 5 min each time, add rabbit secondary antibody and incubate at 37°C for 90 min. DAB staining, hematoxylin re-staining, microscopic observation, and section analysis to evaluate PLPP4 expression.

### Statistical Analysis

For glioma and PAAD patients of The Cancer Genome Atlas (TCGA) database, tumoral RNA-seq data were downloaded from the Genomic Data Commons (GDC) data portal (TCGA) and glioma and PAAD of the tumors also had mRNA expression data of paired normal tissue samples. All values are presented as the mean ± standard deviation (SD). Significant differences were determined using GraphPad 5.0 software (USA). The student’s t-test was used to determine significant differences between two groups. One-way ANOVA was used to determine statistical differences between multiple groups. The chi-square test was used to analyze the relationship between PLPP4 expression and clinicopathological characteristics. Survival curves were plotted using the Kaplan-Meier method and compared by log-rank test. Univariate and multivariate Cox regression analyses were performed using IBM SPSS statistical software (version 24.0), and the clinical value of PLPP4 for survival and prognosis of the two cancers was realized by ROC curve. *P* < 0.05 was considered significant. All the experiments were repeated three times.

## Results

### Expression of LPPs in Different Types of Cancer

**Figure 1:**
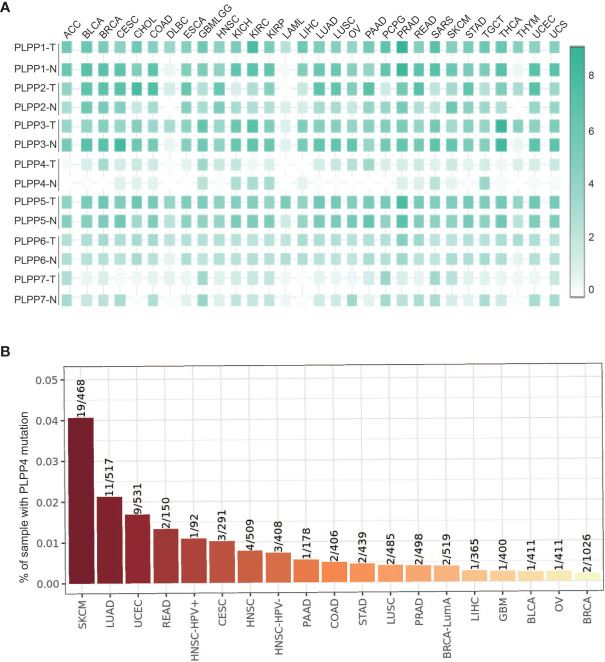
The relative expression status of PLPP4 in cancers. **(A)** Heatmap: the expression patterns of LPPs in cancer (GEPIA). **(B)** Genetic alterations of PLPP4 in cancers (Timer).

We first analyzed the expression levels of LPPs proteins, including PLPP1-7, in the high throughput paired cancers’ RNA expression profile datasets from TCGA and found that the expression levels of PLPP1, PLPP2, PLPP3, PLPP5, PLPP6, and PLPP7 were expressed in both cancer tissues and normal tissues, except PLPP4 (**Figure 1A**). PLPP1 was differentially elevated in lymphoid neoplasm diffuse large B-cell lymphoma (DLBC), acute myeloid leukemia (LAML), and thymoma (THYM). PLPP2 was higher in many cancers like adrenocortical carcinoma (ACC), cholangiocarcinoma (CHOL), colon adenocarcinoma (COAD), pancreatic adenocarcinoma (PAAD), and so on, while the level of PLPP2 was lower in kidney chromophobe (KICH), pheochromocytoma and paraganglioma (PCPG), skin cutaneous melanoma (SKCM), and so on. PLPP3, PLPP4, and PLPP6 were differentially elevated in some cancers, particularly PLPP4 showed “with” and “without” expression in cancer or tumor tissues (T) compared to the respective adjacent normal tissues (N). Conversely, the expression level of PLPP7was decreased to some cancer tissues compared with those in the normal tissues (**Figure 1A**), indicating that different members of the PLPP family have oncogenic or tumor-suppressive roles in the development of cancers. Relatively independently, there was no significant difference in PLPP5 expression between cancer tissues and normal tissues. Next, we want to focus on the analysis of PLPP4 in different cancers because of the specificity of PLPP4 expression between cancer and the respective adjacent normal tissues. Furthermore, A comprehensive analysis of the molecular characteristics of PLPP4 was further performed. As a result, the mutation ratio on PLPP4 was highest in SKCM, and there were showed mutations in LUAD, UCEC, PAAD, and so on (**Figure 1B**). Therefore, our findings indicated that increased expression of PLPP4 was positively associated with advanced clinicopathological features in many cancers.

### Expression of PLPP4 Correlates With Many Cancers

**Figure 2:**
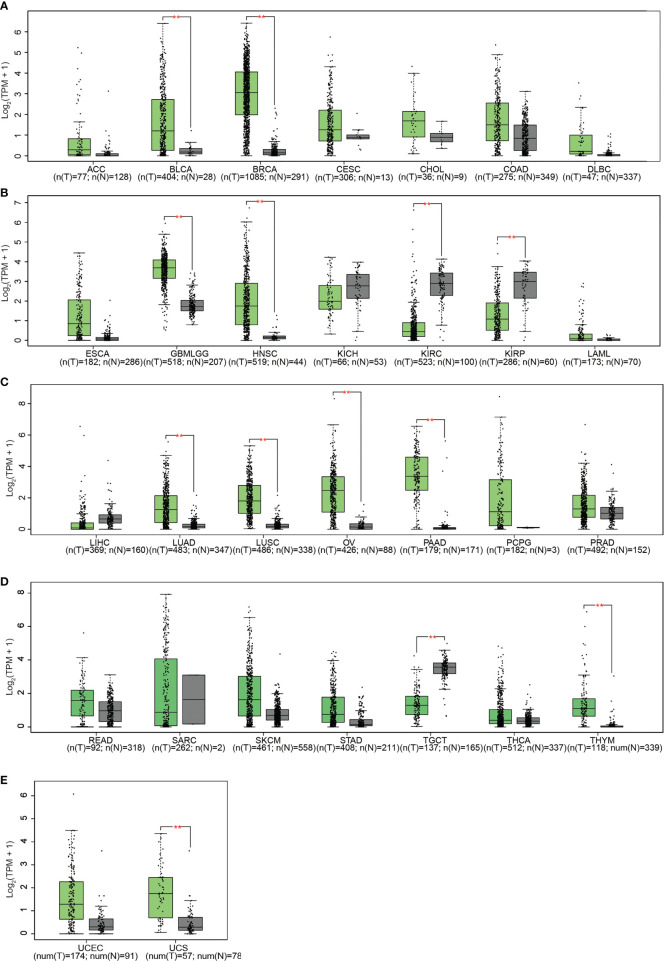
**(A)** The relative expression levels of PLPP4 in ACC, BLCA, BRCA, CESC, CHOL, COAD, DLBC, compared with normal tissues. **(B)** Compared with normal tissues, the relative expression levels of PLPP4 in ESCA, GBMLGG, HNSC, KICH, KIRC, KIRP, LAML. **(C)** Compared with normal tissues, the relative expression levels of PLPP4 in LIHC, LUAD, LUSC, OV, PAAD, PCPG, PRAD. **(D)** Compared with normal tissues, the relative expression levels of PLPP4 in READ, SARC, SKCM, STAD, TGCT, THCA, THYM. **(E)** Compared with normal tissues, the relative expression levels of PLPP4 in UCEC, UCS. "**"means compared to normal, **P < 0.01.

**Figure 3:**
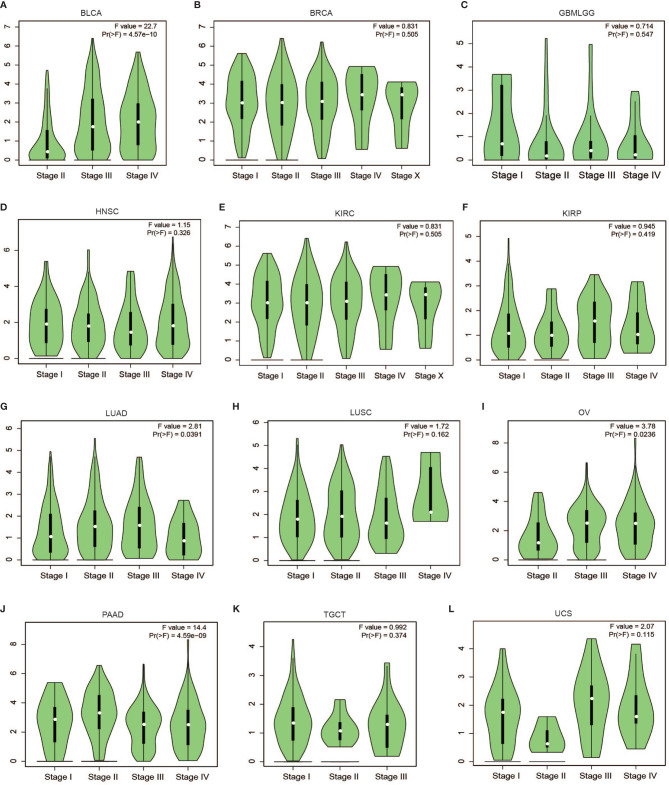
Correlation between PLPP4 and the pathological stage of cancers (GEPIA). There was a significant correlation between PLPP4 expression level and the pathological stage of BLCA, LUAD, OV, and PAAD patients. **(A–C)** PLPP4 expression level and the pathological stage of BLCA, BRCA, and GBMLGG patients. **(D–F)** PLPP4 expression level and the pathological stage of HNSC, KIRC, and KIRP patients. **(G–I)** PLPP4 expression level and the pathological stage of LUAD, LUSC, and OV patients. **(J–L)** PLPP4 expression level and the pathological stage of PAAD, TGCT, and UCS patients.

Subsequent analysis of PLPP4 expression in different types of cancer datasets from TCGA and ArrayExpress showed that PLPP4 expression was upregulated in BLCA, BRCA, GBMLGG, HNSC, LUAD, LUSC, OV, PAAD, TGCT, THYM, and UCS tissues, and down-regulated in KIRC and KIRP compared that in the respective adjacent normal tissues and was further increased in LGG and PAAD tissues (**Figures 2A–E**). We then assessed the correlation between the expression of PLPP4 and the pathological stage of BLCA, BRCA, GBMLGG, HNSC, KIRC, KIRP, LUAD, LUSC, OV, PAAD, TGCT, THYM, and UCS patients. We found a significant correlation between the expression of PLPP4 and pathological stage in BLCA, LUAD, OV, and PAAD patients (**Figures 3A** **–L**). These data suggested that PLPP4 played significant roles in developing BLCA, LUAD, OV, and PAAD.

**Figure 4:**
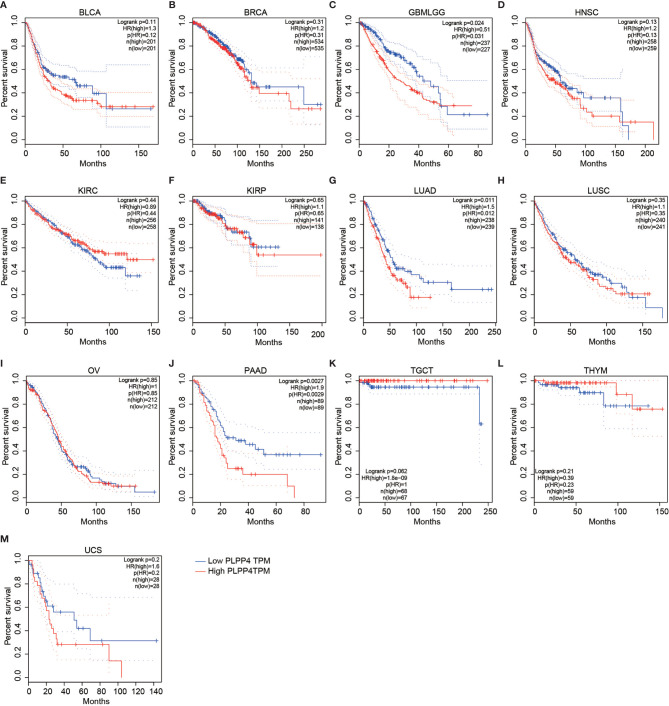
Overall survival curves for the expression of PLPP4 in cancers (Kaplan–Meier Plotter). High expression level of PLPP4 indicated shorter overall survival in glioma, LUAD, and PAAD cases. **(A–D)** Survival curves for the expression of PLPP4 in BLCA, BRCA, GBMLGG and HNSC. **(E–H)** Survival curves for the expression of PLPP4 in KIRC, KIRP, LUAD and LUSC. **(I–L)** Survival curves for the expression of PLPP4 in OV, PAAD, TGCT, and THYM. **(M)** Survival curves for the expression of PLPP4 in UCS.

To investigate the clinical correlation of PLPP4 with survival in BLCA, BRCA, GBMLGG, HNSC, KIRC, KIRP, LUAD, LUSC, OV, PAAD, TGCT, THYM, and UCS patients, these datasets from TCGA, ArrayExpress, and Kaplan-Meier Plotter were further analyzed. The results revealed that GBMLGG, LUAD, and PAAD patients with high expression of PLPP4 exhibited shorter overall survival rates compared with the corresponding patients with “low” or “none” expression of PLPP4 (**Figures 4C** **, G, J**). Meanwhile, there was no significant difference in the clinical correlation of PLPP4 with survival in other cancers (**Figure 4**). Collectively, these results from publicly available cancer datasets suggest that the overexpression of PLPP4 correlates with poor prognosis and progression status in glioma, LUAD, and PAAD patients.

### Prognostic Value of Single CpG of PLPP4 in Cancers

**Figure 5:**
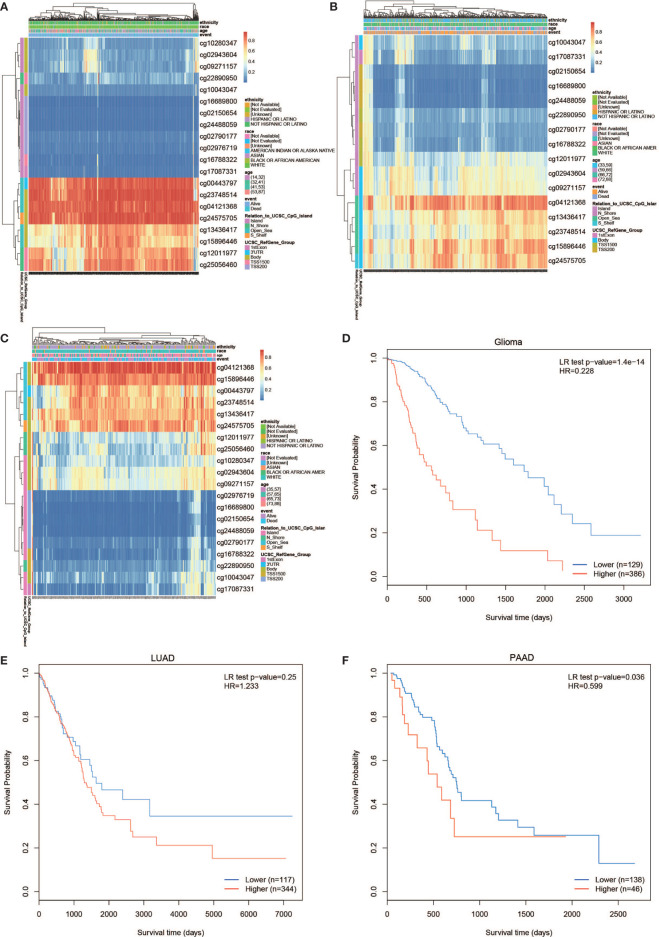
The prognostic value of the DNA methylation of PLPP4 signature in glioma, LUAD, and PAAD *via* SurvivalMeth. **(A)** The heat map of DNA methylation clustered expression level of PLPP4 in glioma. **(B)** The heat map of DNA methylation clustered expression level of PLPP4 in LUAD. **(C)** The heat map of DNA methylation clustered expression level of PLPP4 in PAAD. Notes: cg04121368 of PLPP4 showed the highest DNA methylation level in glioma, LUAD, and PAAD. **(D–F)** The survival curve of the Kaplan–Meier plot.

The PLPP4 mRNA was used for prognostic analysis in SurvExpress. The prognostic value of DNA methylation of PLPP4 in glioma, LUAD, and PAAD was analyzed by MethSurv. The heat maps of DNA methylation of PLPP4 are displayed in **Figures 5A** **–C**. Among them, cg04121368 of PLPP4in glioma, LUAD, and PAAD showed the highest DNA methylation level. And overall, we found that 15CpGs of PLPP4 in glioma, 3CpGs of PLPP4 in PAAD were significantly associated with prognosis in GLIOMA and PAAD patients, but no significant association in LUAD patients (**Table 1**). The DNA methylation level was significantly associated with survival probability in glioma and PAAD patients. However, no statistically significant association in LUAD was found between the high- and low-risk groups (**Figures 5D** **–F**). Therefore, our results indicated that PLPP4 might be implicated in the development and progression of glioma and PAAD.

**Table 1:** The Prognostic Value of Single CpG of PLPP4 in Glioma and PAAD by MethSurv (P < 0.05).

Name	Cancer	HR	LR_test_pvalue	UCSC_RefGene_Group	Relation_to_UCSC_CpG_Island
cg00443797	GBMLGG	0.228	1.39888E-14	3'UTR	Open_Sea
cg02943604	GBMLGG	1.931	0.000559886	Body	Island
cg02976719	GBMLGG	1.472	0.031558747	TSS200	Island
cg09271157	GBMLGG	2.871	3.78503E-08	Body	Island
cg10280347	GBMLGG	2.852	2.7388E-08	Body	Island
cg12011977	GBMLGG	0.259	1.01985E-12	TSS1500	N_Shore
cg13436417	GBMLGG	0.373	7.18685E-08	Body	Open_Sea
cg15896446	GBMLGG	0.473	0.000143785	Body	Open_Sea
cg16689800	GBMLGG	1.639	0.016499818	TSS200	Island
cg17087331	GBMLGG	1.76	0.003755979	1stExon	Island
cg22890950	GBMLGG	0.31	1.51908E-09	TSS1500	N_Shore
cg23748514	GBMLGG	0.266	8.90499E-12	Body	Open_Sea
cg24488059	GBMLGG	1.522	0.039639294	TSS200	Island
cg24575705	GBMLGG	0.333	4.78143E-08	Body	S_Shelf
cg25056460	GBMLGG	0.234	2.86438E-14	TSS1500	N_Shore
cg02943604	PAAD	1.647	0.045274299	Body	Island
cg09271157	PAAD	1.677	0.031896675	Body	Island
cg15896446	PAAD	0.599	0.035879869	Body	Open_Sea

### Identification and Establishment of PLPP4 Prognostic Signature in Glioma

**Figure 6:**
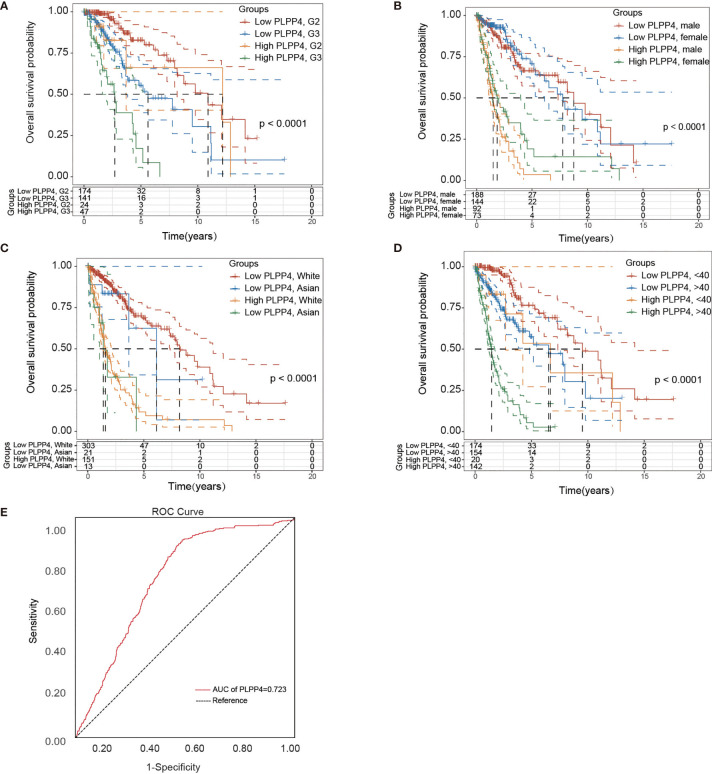
Over survival and ROC analysis of PLPP4 signature risk score in glioma. **(A–D)** Over survival analysis based on the effect of tumor grade, gender, race, and age. **(E)** ROC analysis of the sensitivity and specificity of the prognosis prediction based on overall survival. AUC, area under the ROC curve.

Univariate and Multivariate Cox regression analyses were applied to identify overall survival-related differentially expressed genes and establish a prognostic gene signature whose performance was evaluated by Kaplan-Meier curve, receiver operating characteristic (ROC). PLPP4 was significantly associated with overall survival based on the univariate Cox regression model (*P* < 0.001, **Table 2**). Multivariate Cox analysis showed that PLPP4 expression level (HR=1.041, 95%CI: 0.561~1.774, P=0.011), WHO grade, age, and lymph node invasion and metastasis influenced poor factors prognosis of glioma patients (**Table 2**). Based on the expression level of PLPP4, we performed a subgroup analysis of tumor grade, age, race, gender, recurrence, and metastasis for glioma. Further, we analyzed the impact of different subgroups on survival and prognosis (**Figures 6A** **–D**). We found that tumor grade, age, race, and gender subgroups impacted the prognosis of glioma (all <0.001). ROC curve analysis was used to analyze the diagnostic effect of PLPP4 expression on glioma patients. The analysis results showed that the area under ROC curve analysis (AUC) of PLPP4 expression in predicting glioma was 0.723 (95%CI: 0.696 ~ 0.749, *P* < 0.001), its sensitivity and specificity were 89.8% and 51.3%, as shown in **Figure 6E**. In general, the PLPP4 signature performed well at predicting the overall survival of glioma.

**Table 2:** Risk factors for survival and prognosis of glioma patients by COX proportional hazards model using TCGA date.

Indicators	Single Factor		Multivariate Factor	
	HR (95% CI)	p-value	HR (95% CI)	p-value
Age (>60 years/<60 years)	0.229 (0.166-0.316)	<0.001	1.056 (0.833-1.295)	<0.001
Gender (male/famale)	1.216 (0.941-1.571)	0.135	1.149 (0.719-1.834)	0.562
Stage (T1/T2/T3)	0.294 (0.199-0.435)	<0.001	0.245 (0.127-0.474)	<0.001
Metastasis (NO/YES)	1.297 (0.706-12.383)	0.401	1.063 (0.716-1.61)	<0.001
PLPP4expression (High/Low)	1.492 (1.141-2.262)	<0.001	1.041 (0.561-1.774)	0.011

### Identification and Establishment of PLPP4 Prognostic Signature in PAAD

**Figure 7:**
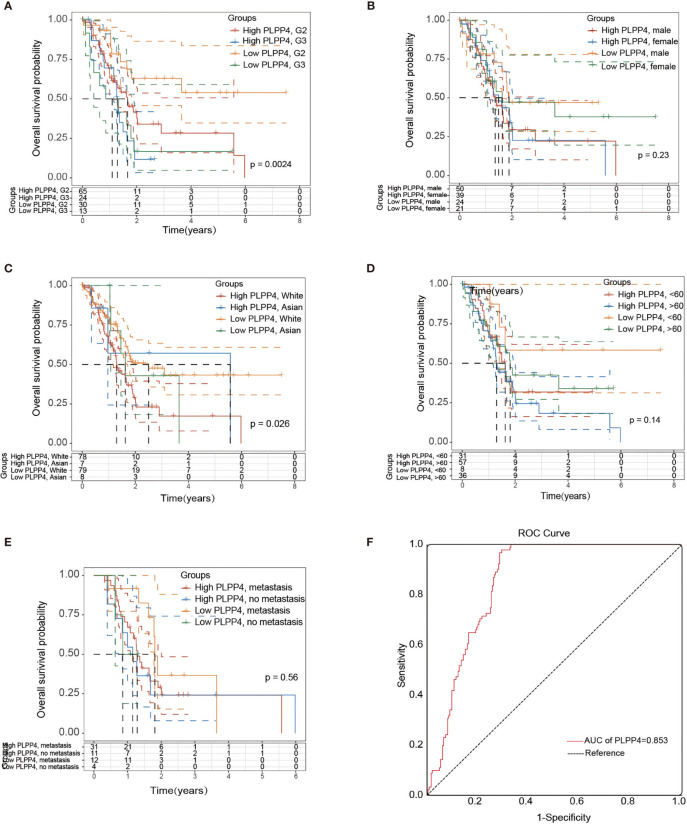
Over survival and ROC analysis of PLPP4 signature risk score in PAAD. **(A–E)** Influence of grade, gender, race, and metastasis on over survival with high or low PLPP4 expression. **(F)** ROC analysis of the sensitivity and specificity of the prognosis prediction based on overall survival. AUC, area under the ROC curve.

More than one hundred sixty patients from the TCGA PAAD dataset were included in subsequent survival analyses. The expression of PLPP4 in PAAD with age, sex, lymph node invasion and metastasis, TNM stage, and other clinical traits was analyzed by univariate Cox regression analysis. The results indicated that the stage of PAAD (HR 0.481, P=0.034) and the expression of PLPP4 (HR 1.767, P=0.007) were associated with prognostic survival. Multivariate Cox regression analysis showed that age (HR 0.366, P=0.008), stage (HR 1.614, P=0.031), and expression of PLPP4 (HR 1.766, P=0.008) were correlated with prognosis and survival. This suggests that PLPP4 overexpression is an independent poor prognostic factor for PAAD (**Figure 7** and **Table 3**). Based on the expression level of PLPP4, we performed a subgroup analysis of PAAD based on tumor grade, age, race, gender, recurrence, and metastasis. Further, we analyzed the impact of different subgroups on survival and prognosis (**Figure 7A–E**). The subgroup analysis of tumor grade (P=0.002) and race (P=0.026) was significant (**Figures 7A** **–C**). Subsequently, we conducted ROC analyses to assess how PLPP4 could behave in predicting prognosis. As shown in **Figure 7F**, the area under the ROC curve (AUC) of the PLPP4 risk score model performed on overall survival in the training cohort was 0.853 (95%CI: 0.814-0.892, *P* < 0.001), and its sensitivity and specificity were 97.8% and 70.7%. Consistently, in the prediction model of progression-free survival predicted in the training cohort, PLPP4 signature risk score also showed a powerful ability. In general, PLPP4 signature performed well at predicting the overall survival of pancreatic cancer.

**Table 3:** Risk factors for survival and prognosis of pancreatic cancer patients by COX proportional hazards model using TCGA date.

Indicators	Single Factor		Multivariate Factor	
	HR (95% CI)	p-value	HR (95% CI)	p-value
Age (>60 years/<60 years)	0.705 (0.445-1.118)	0.137	0.366 (0.174-0.767)	0.008
Gender (male/famale)	0.809 (0.537-1.219)	0.311	1.019 (0.584-1.777)	0.947
Stage (T1/T2/T3)	0.481 (0.245-0.944)	0.034	1.614 (1.045-2.495)	0.031
Metastasis (NO/YES)	0.634 (0.352-1.139)	0.127	0.909 (0.4-2.064)	0.820
PLPP4 expression (High/Low)	1.767 (1.166-2.677)	0.007	1.766 (1.161-2.686)	0.008

### Silencing PLPP4 Abrogates the Proliferation Ability of Glioma Cells

**Figure 8:**
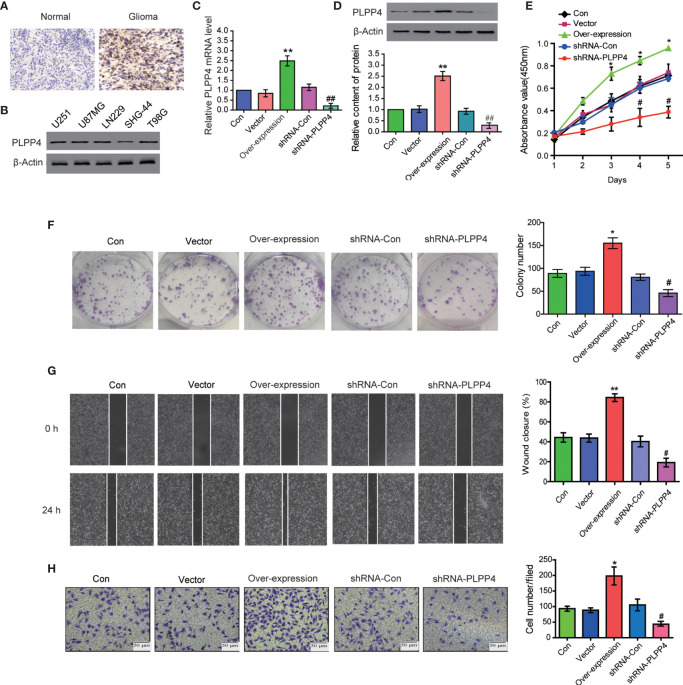
PLPP4 promoted the proliferation, migration, and invasion of glioma LN229 cells. **(A)** Representative image of immunohistochemical analysis of paraffin-embedded primary glioma tissue specimens (enlarged X400). PLPP4 staining was stronger in glioma tissues than in normal tissues with a scale of 20μm. **(B)** PLPP4 expression in different glioma cell lines. **(C)** Real-time PCR assay was used to detect the expression of PLPP4 (vector, over-expression, shRNA-con, shRNA-PLPP4) in each group of LN229 cells. **(D)** Western blot analysis was performed to detect PLPP4 expression (vector, over-expression, shRNA-con, shRNA-PLPP4) in each group of LN229 cells. **(E)** CCK-8 assay (at 1, 2, 3, 4, 5 days) in LN229cells. PLPP4-interference inhibited the proliferation of LN229 cells. **(F)** Silencing PLPP4 reduced the mean colony number according to the colony formation assay. **(G)** The migration of LN229 cells in each group was detected by the scratch method. **(H)** In invasion assay, representative images of LN229 cells (vector, over-expression, shRNA-con, shRNA-PLPP4) (×100). Each bar represents the mean values ± SEM of three independent experiments. Compared to vector, *P < 0.05, **P < 0.01; compared to shRNA-Con, ^#^ *P* < 0.05, ^##^ *P* < 0.01.

PLPP4 is widely expressed in various tumor cells and plays a role in tumor genesis and invasion. PLPP4 promotes proliferation and tumorigenesis *via* activating the influx of intracellular Ca^2+^ in lung adenocarcinoma (ADC) tissues (13, 24). To explore the biological roles of PLPP4 in glioma, immunohistochemical analysis of PLPP4 expression in glioma tissues was further examined (**Figure 8A**). As shown in **Figure 8A**, PLPP4 expression was primarily detected in the cytoplasm, and the staining intensity of PLPP4 was increased in glioma tissues compared with normal tissues. Therefore, our findings indicated that high expression of PLPP4 was positively associated with advanced clinicopathological features in glioma patients. Then we first examined PLPP4 expression levels in glioma cell lines by western blot, and LN229 of the glioma cell line was used for the further experiment (**Figure 8B**). As shown in **Figures 8C** **, D**, the mRNA and protein levels of PLPP4 were differentially increased *via* overexpression compared with vector.

In contrast, the mRNA and protein levels of PLPP4 were differentially decreased *via* sh-RNA compared with sh-con. To investigate the effects of overexpression or silencing of PLPP4 on proliferation, migration, and invasion of glioma LN229 cells, we first used CCK-8 cell proliferation assay to confirm that overexpression of PLPP4 enhanced cell proliferation and increased with time (P<0.05). Knocking down PLPP4 inhibited cell proliferation (**Figure 8E**). Colony formation assays revealed that silencing PLPP4 dramatically inhibited the colony-forming ability of LN229 cell; while PLPP4 overexpression promoted the growth of LN229 cell (**Figure 8F**). The scratch test determined the effect of PLPP4 on the migration of LN229. The results showed that overexpression of PLPP4 significantly increased the mobility of LN229 cells, while silencing of PLPP4 significantly decreased the mobility of LN229 cells (**Figure 8G**). Recent studies have found that PLPP4 can promote the invasion and migration in LUAD and gastric cancer (13, 25). Our results also showed that overexpression of PLPP4 promoted the invasiveness of glioma LN229 cells, while the number of cells in the shRNA-PLPP4 group was reduced, and the invasiveness was reduced by 55.2% through the transwell assay (**Figure 8H**). The results showed that inhibiting PLPP4 expression could effectively reverse the proliferation, migration, and invasion of glioma LN229 cells. Taken together, high expression of PLPP4 correlated with advanced proliferation.

### Correlation Analysis of PLPP4 With Other Genes in Glioma

**Figure 9:**
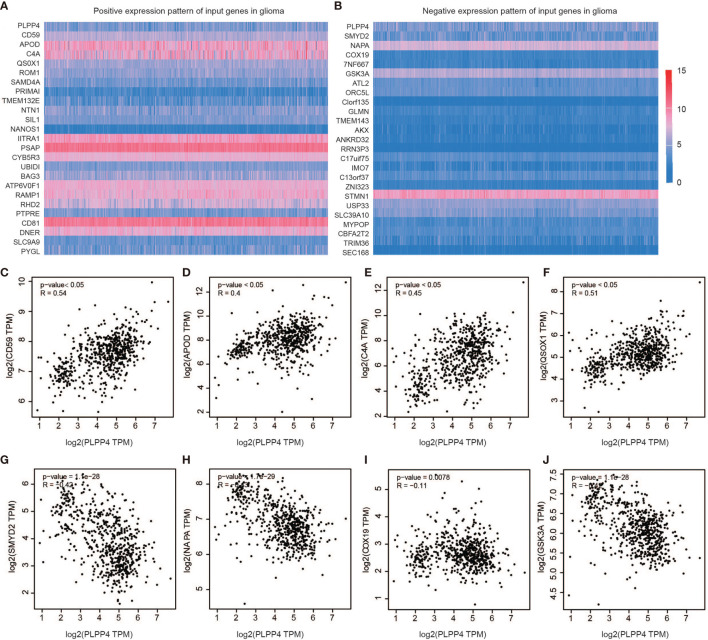
Genes correlated with PLPP4 in glioma. **(A)** Genes positively correlated with PLPP4 in glioma **(B)** Genes negatively linked with PLPP4 in glioma. **(C–F)** Positive correlation analysis between PLPP4 and other genes (CD59, APOD, C4A, QSOX1). **(G–J)** Negative correlation analysis between PLPP4 and other genes (SMYD2, NAPA, COX19, GSK3A).

Further, we analyzed the correlation between PLPP4 and other genes, and the heatmap showed genes that are positively correlated with PLPP4 and genes which are negatively associated with PLPP4 in glioma (**Figures 9A** **, B**). Correlogram analysis also found CD59, APOD, C4A, and QSOX1 were upregulated, while SYMD2, NAPA, COX19, and GSK3A were down-regulated (**Figures 9C** **–J**). These data were consistent with the heatmap results.

**Figure 10:**
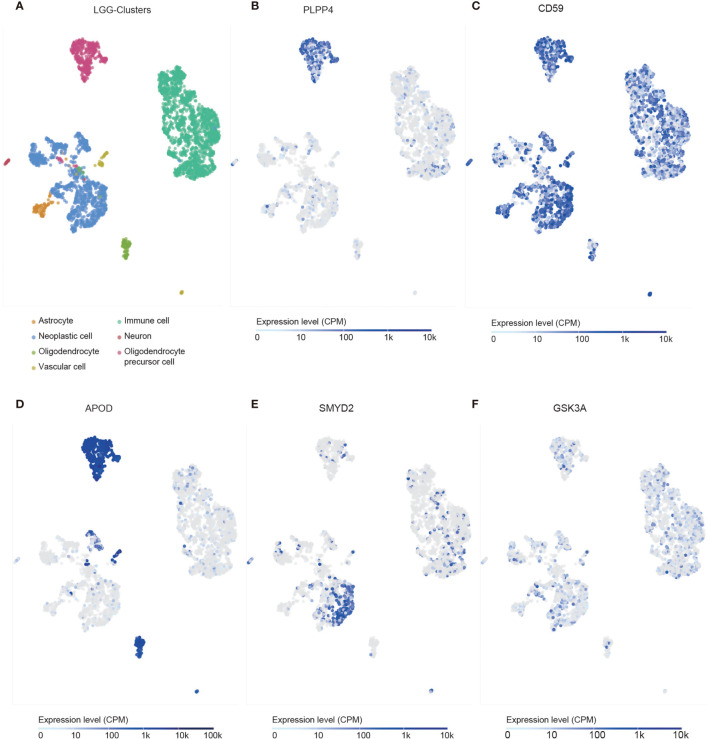
Single-cell RNAseq analysis of PLPP4 and other genes at the migrating front of human glioma. **(A)** Major cell groups were identified for glioma. The cells were categorized into 7 clusters. **(B–F)** Co-expression of PLPP4 and other genes in glioma.

We identified the specificity of PLPP4 expression in glioma by single-cell sequencing analysis. PLPP4 was mainly expressed in oligodendrocyte precursor cells, a type of glial cell, and immune cells also detected the PLPP4 expression (**Figures 10A** **, B**). To further explore the research mechanism, we determined the co-expression of PLPP4 and other genes (**Figures 10C** **–F**). Positive correlation genes, like CD59 and APOD, had high co-expression, while negative correlation genes, like SMTD2 and GSK3A, had low co-expression. APOD and PLPP4 were mainly expressed in oligodendrocyte precursor cells. Most of these genes were related to the regulation of apoptosis signaling pathway, inflammatory process, and cellular metabolic pathway (26, 27). CD59 is highly expressed in several cancer cell lines and tumor tissues and regulates the function, infiltration, and phenotypes of various immune cells in the tumor microenvironment (28). APOD may be a potential therapeutic target for tumor angiogenesis by suppressing PI3K-Akt-eNOS signaling, an essential pathway regulating angiogenesis (29, 30).

### Silencing PLPP4 Abrogates the Proliferation Ability of PAAD Cells

**Figure 11:**
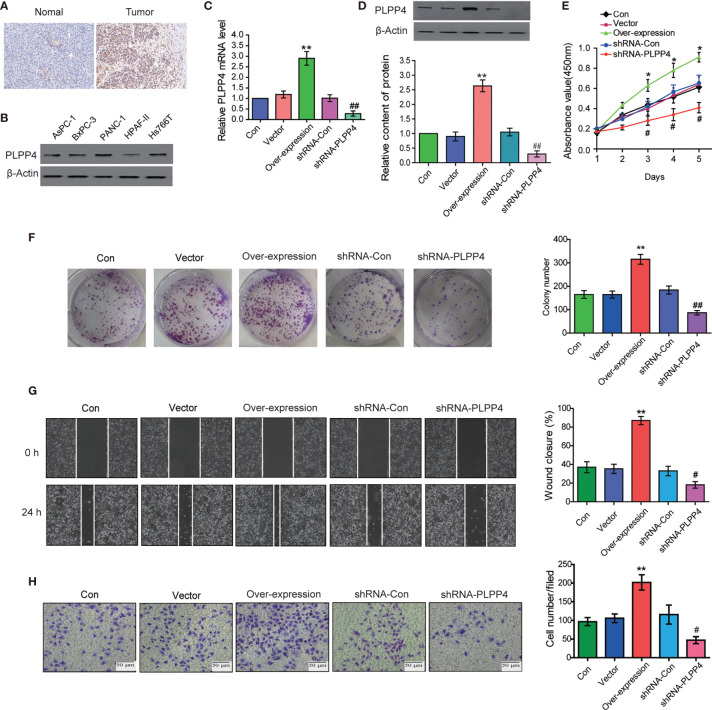
Silencing PLPP4 decreased the proliferation, migration, and invasion of pancreatic cancer cells. **(A)** Expression of PLPP4 protein in pancreatic cancer, a representative image of immunohistochemical analysis of paraffin-embedded specimens from 65 cases of primary pancreatic cancer. **(B)** Western blot was used to detect the expression of PLPP4 protein in different pancreatic cancer cell lines. **(C)** The real-time PCR assay was used to detect the expression of PLPP4(vector, over-expression, shRNA-con, shRNA-PLPP4) in PANC-1 cells. **(D)** Western blot analysis of PLPP4 expression in PANC-1 cells of pancreatic cancer. **(E)** PANC-1 cells proliferation was analyzed with the CCK-8 assay at 1, 2, 3, 4, 5 days. **(F)** Silencing PLPP4 reduced the mean colony number in PANC-1 cells according to the colony formation assay. **(G)** shRNA-PLPP4 or over-expression PLPP4 was transfected into PANC-1 cells, and the migration of cells in each group was detected by the scratch method. **(H)** Invasion assay was used to detect the invasion of PANC-1 cells. Each bar represents the mean values ± SEM of three independent experiments. Compared to vector, *P < 0.05, **P < 0.01; compared to shRNA-Con, ^#^ *P* < 0.05, ^##^ *P* < 0.01.

PLPP4 is widely expressed in a variety of tumor cells and plays a role in tumor genesis and invasion. Another cancer with a low survival rate, PAAD, was analyzed in this project. First, immunohistochemistry showed that the expression of PLPP4 protein in PAAD tissues was significantly higher than that in normal pancreatic cancer tissues (**Figure 11A**). As the expression of PLPP4 was detected in various pancreatic cancer cell lines (**Figure 11B**), we constructed PLPP4-stably suppressing PANC-1 cells by endogenously knocking down PLPP4 *via* retroviral infection; meanwhile, we transfected of the constructed plasmid (vector, over-expression) into pancreatic cancer PANC-1 cells (**Figures 11C** **, D**). CCK-8 assays were carried out, and the results showed downregulation of PLPP4 decreased viability in PANC-1 cells (**Figure 11E**). Overexpression of PLPP4 significantly enhanced the proliferation, migration, and invasion of PANC-1 cells. Inhibition of PLPP4 expression could down-regulate the proliferation, migration, and invasion of PANC-1 cells (**Figures 11F** **–H**). Taken together, these findings demonstrated that silencing PLPP4 inhibits the proliferation ability of PANC-1 cells.

### Correlation Analysis of PLPP4 With Other Genes in PAAD

**Figure 12:**
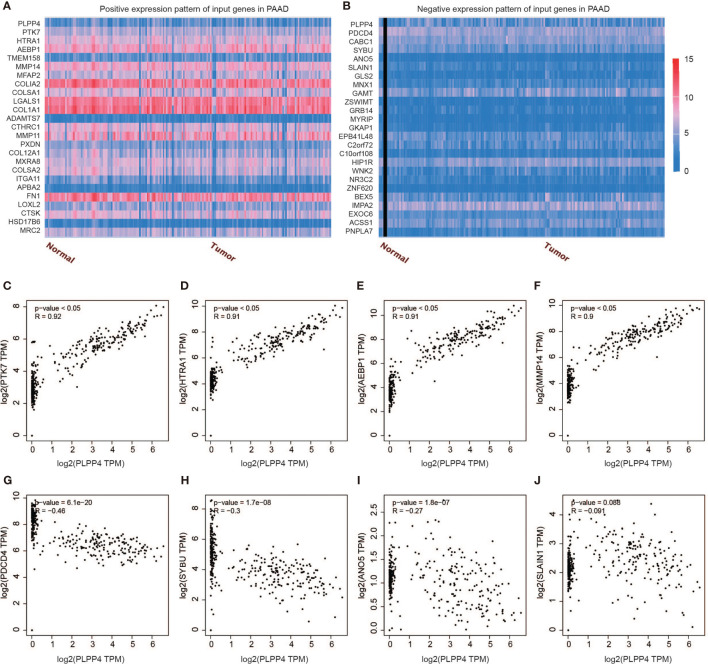
Genes correlated with PLPP4 in PAAD. **(A)** Genes positively correlated with PLPP4 in PAAD **(B)** Genes negatively related with PLPP4 in PAAD. **(C–F)** Positive correlation analysis between PLPP4 and other genes (PTK7, HTRA1, AEBP1, MMP14). **(G–J)** Negative correlation analysis between PLPP4 and other genes (PDCD4, SYBU, ANO5, SLAIN1).

Further, we analyzed the correlation between PLPP4 and other genes, and the heatmap showed genes that are positively correlated with PLPP4 and genes which are negatively associated with PLPP4 in PAAD (**Figures 12A** **, B**). Correlogram analysis also found PTK7, HTRA1, AEBP1, and MMP14 were upregulated, while PDCD4, SYBU, ANO5, and SLAIN1 were down-regulated (**Figures 12C** **–J**). These data were consistent with the heatmap results. Among them, PTK7 promotes cell migration by regulating Wnt signaling pathway (31, 32). MMP14 acts as a positive regulator of cell growth and migration *via* activation of MMP15 (33). PDCD4 (programmed cell death protein 4) inhibits tumor promoter-induced neoplastic transformation (34, 35). These analyses could explain the short survival and high mortality of PAAD.

### Immune Cell Infiltration of PLPP4 in Patients With Cancers

**Figure 13:**
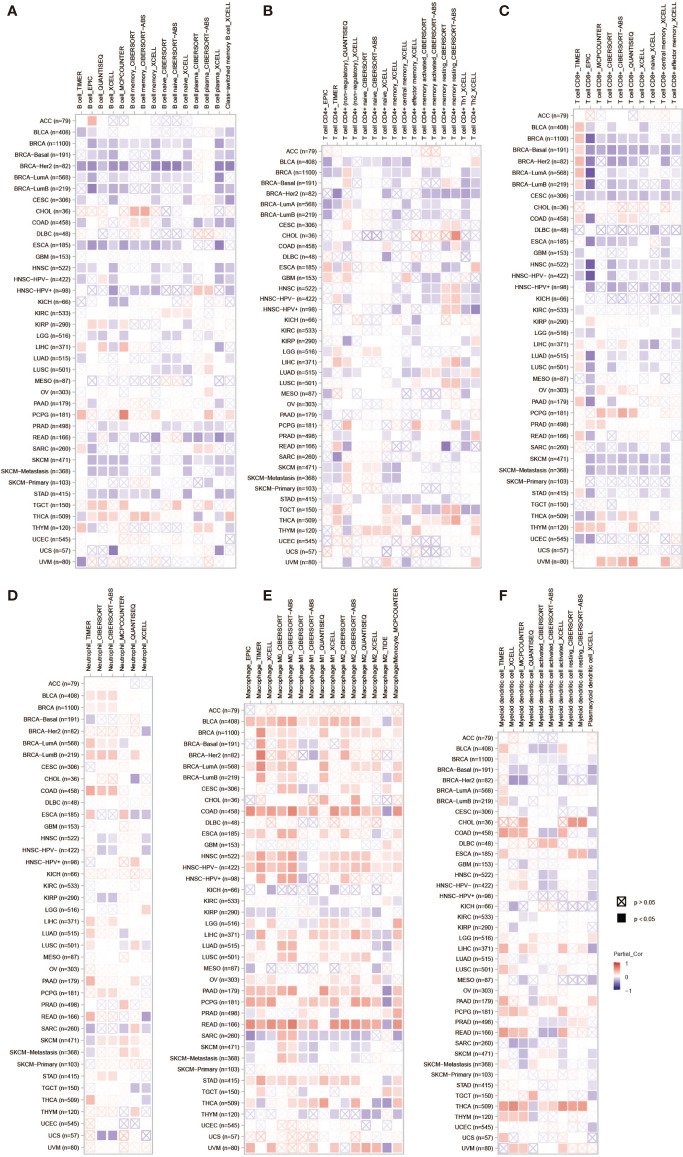
The correlation between PLPP4 and immune cell infiltration in cancers. A comprehensive analysis of the correlation between PLPP4 and six immune cell infiltrations (B cells, CD4+ T cells, CD8+ T cells, neutrophils, macrophages, and dendritic cells) was performed using TIMER web server. **(A)** The correlation between PLPP4 and B cells infiltrations. **(B)** The correlation between PLPP4 and CD4+ T cells infiltrations. **(C)** The correlation between PLPP4 and CD8+ T cells infiltrations. **(D)** The correlation between PLPP4 and neutrophils infiltrations. **(E)** The correlation between PLPP4 and macrophages infiltrations. **(F)** The correlation between PLPP4 and dendritic cells infiltrations.

PLPP4 has been involved in cancer-related inflammation and the infiltration of immune cells, thus affecting the clinical outcome of cancers. Therefore, the TIMER database was used to comprehensively analyze the correlation between PLPP4 and immune cell infiltration. The results are presented in **Figure 13**. PLPP4 was positively correlated with infiltration of two immune cell types (macrophages and dendritic cells; all *P* < 0.05). Our results further verify that overexpression of PLPP4 is observed in the glioma and PAAD tissues and cells and positively correlates with advanced clinicopathological features and poor prognosis in glioma and PAAD patients. Silencing PLPP4 inhibits the proliferation and tumorigenicity of glioma and PAAD cells *in vitro*. In addition, our findings reveal that DNA methylation of PLPP4 is related to survival probability in glioma and PAAD patients. Taken together, our findings indicate that PLPP4 plays an important role in the progression of glioma and PAAD and suggest that PLPP4 may serve as a potential target for human glioma and PAAD treatment.

## Discussion

Glioma and pancreatic cancer are prevalent, highly malignant, and highly lethal forms with abysmal prognosis. Accurate prognosis prediction can identify patients benefiting from more radical treatment, including neoadjuvant therapy, more intensive surgery, chemotherapy, radiation therapy, targeted molecular medicine, and immunotherapy. More and more gene targets were found in tumors as research progressed, also including non-coding RNA. Circular RNA-encoded oncogenic E-cadherin variant promoted glioblastoma tumorigenicity through activation of EGFR–STAT3 signaling (36). Genes associated with immunity, like IL4I1, CCR5, CD73, CD39, and so on. IL4I1 is a metabolic, immune checkpoint that activates the aryl hydrocarbon receptor and promotes glioma progression (37, 38). Zhang et al. reported that silencing the CCL5-CCR5 signaling largely abrogated the glioma-protective effects of pericytes and enhanced the chemotherapeutic efficacy of temozolomide (39). CD73 and CD39 as combinatorial targets are the specific immunotherapeutic targets to improve anti-tumor immune responses in glioma (40, 41). These findings demonstrate that comprehensive human and reverse translational studies can be used for the rational design of combinatorial immune checkpoint strategies.

Another type of cancer with a poor prognosis, PAAD, was also tested for some genes associated with prognosis including proteins, non-coding RNA, and genetic modification. MET, KLK10, COL17A1, CEP55, ANKRD22, ITGB6, ARNTL2, MCOLN3, and SLC25A45 were established to predict the overall survival of PAAD (42, 43). Emerging evidence suggests that competing endogenous RNAs play a crucial role in developing and progressing pancreatic adenocarcinoma (PAAD). The objective was to identify a new lncRNA-miRNA-mRNA network as prognostic markers and develop and validate a multi-mRNAs-based classifier for predicting overall survival in PAAD (44). The expression of CXCR4, HIF1A, ZEB1, and SDC1 in PAAD was regulated by circ-UBAP2 and hsa-miR-494 (45). N6-methyladenosine (m6A) has an important epitranscriptomic modification that controls PAAD self-renewal and cell fate. M6A-related genes like HNRNPC, IGF2BP2, and YTHDF1, are differentially expressed between PAAD and normal tissues (46, 47). Our study also found that the level of DNA methylation of PLPP4 was related to the survival of glioma and PAAD (**Figure 5**).

In this study, we first investigated the expression of LPPs in the glioma and PAAD and found PLPP4 was over-expressed in the glioma and PAAD compared with the normal tissues. Besides, the relationship between PLPP4 and the pathological stage of the glioma and PAAD was assessed; we found the expression of PLPP4 decreased as the adenocarcinomas progressed in PAAD. But there was no significant difference in glioma patients. Furthermore, we investigated the prognostic values of PLPP4 in the glioma and PAAD cases. The results showed that high expression of PLPP4 was significantly associated with worse OS. And for the prognostic value of the DNA methylation of PLPP4, 15 CpGs of PLPP4, 3 CpGs of PLPP4 were significantly associated with prognosis in glioma and PAAD patients. We further performed Immunohistochemical analysis to investigate the effects of PLPP4 on the tumorigenic activity of the glioma and PAAD patients and found that PLPP4 showed high expression in the glioma and PAAD patients. Silencing PLPP4 reduced the proliferation by LN229 and PANC-1 cells and significantly decreased the migration and invasion (**Figures 6**, **9**). Taken together, these findings demonstrated that silencing PLPP4 inhibits the proliferation, migration, and invasion ability of the glioma and PAAD cells. Furthermore, we also predicted the molecular mechanism associated with PLPP4 in tumor progression.

Moreover, the correlation between PLPP4 and immune cell infiltration of cancers was also assessed. PLPP4 was significantly related to all six immune cell types, including B cells, CD8+ T cells, CD4+ T cells, macrophages, neutrophils, and dendritic cells. And there was a significant positive correlation between PLPP4 expression and the infiltration of macrophages. These results indicated that PLPP4 was involved in the glioma and PAAD progression by affecting immune status.

PLPP4 expression levels of LPPs in glioma and PAAD were statistically different from normal tissues according to the results of **Figures 1** and **2**. Meanwhile, PLPP4 was considered as a prognostic biomarker because PLPP4 showed “no-expression” or “expression” in normal tissues and carcinoma tissues, like ACC, BLCA, BRCA, DLBC, ESCA, HNSC, LAML, LUAD, LUSC, OV, PAAD, THYM, and so on (**Figures 1A** and **2**). In addition, the expression levels of PLPP4 in glioma were also higher than normal tissues. The correlation between PLPP4 and pathological tumor stages (GEPIA) analysis in **Figure 3** showed that the expression level of PLPP4 was significantly correlated with the pathological stages of PAAD. While **Figures 4**–**7** showed that high PLPP4 expression suggested a shorter overall survival of patients with glioma and PAAD. In conclusion, PLPP4 is highly expressed in PAAD and glioma. In clinical disease research, pancreatic cancer is known as the “king of cancer”, early diagnosis is difficult, the treatment effect is poor, and the fatality rate remains high. Glioma mainly grows in an invasive manner, with rapid diffusion and short survival. In the current study, we first revealed that PLPP4 was elevated in the glioma and PAAD. High expression of PLPP4 significantly correlated with advanced clinicopathological features and poor overall and progression-free survival in the glioma and PAAD patients. In conclusion, this work provided evidence of the values of PLPP4 as clinical biomarkers and therapeutic targets in glioma and PAAD. We hope the results could afford some new inspirations for immunotherapeutic drug development, provide some assistance to the clinicians in the selection of optimal drugs for the glioma and PAAD patients, and identify the tumor markers that have more accurate prognostic prediction ability in the glioma and PAAD.

## Data Availability Statement

The datasets presented in this study can be found in online repositories. The names of the repository/repositories and accession number(s) can be found in the article/supplementary material.

## Ethics Statement

The studies involving human participants were reviewed and approved by the Medical Ethics Committee of Zibo Central Hospital. The patients/participants provided their written informed consent to participate in this study.

## Author Contributions

HW and JD designed the research and wrote the manuscript. WT, ZW, and PW performed analysis and analyzed results and data. ZW and HQ contributed research materials. All authors contributed to the article and approved the submitted version.

## Funding

This work was financially supported by the National Natural Science Foundation of China (81602327 and 81500798) and the Funds for Zhishan Young Scholars (Southeast University; 2242021R41070).

## Conflict of Interest

The authors declare that the research was conducted in the absence of any commercial or financial relationships that could be construed as a potential conflict of interest.

## Publisher’s Note

All claims expressed in this article are solely those of the authors and do not necessarily represent those of their affiliated organizations, or those of the publisher, the editors and the reviewers. Any product that may be evaluated in this article, or claim that may be made by its manufacturer, is not guaranteed or endorsed by the publisher.

## Abbreviations

ACC, adrenocortical carcinoma; BLCA, bladder urothelial carcinoma; BRCA, breast invasive carcinoma; CESC, cervical squamous cell carcinoma and endocervical adenocarcinoma; CHOL, cholangiocarcinoma; COAD, colon adenocarcinoma; DFS, disease-free survival; DLBC, lymphoid neoplasm diffuse large B-cell lymphoma; ESCA, esophageal carcinoma; GBM, glioblastoma multiforme; GBMLGG, glioma; HNSC, head and neck squamous cell carcinoma; KICH, kidney chromophobe; KIRC, kidney renal clear cell carcinoma; KIRP, kidney renal papillary cell carcinoma; LAML, acute myeloid leukemia; LGG, brain lower grade glioma; LIHC, liver hepatocellular carcinoma; LUAD, lung adenocarcinoma; LUSC, lung squamous cell carcinoma; OV, ovarian serous cystadenocarcinoma; PAAD, pancreatic adenocarcinoma; PCPG, pheochromocytoma and paraganglioma; PLPP4, phospholipid phosphatase 4; PRAD, prostate adenocarcinoma; READ, rectum adenocarcinoma; SKCM, skin cutaneous melanoma; STAD, stomach adenocarcinoma; TGCT, testicular germ cell tumors; THCA, thyroid carcinoma; THYM, thymoma; UCEC, uterine corpus endometrial carcinoma; UCS, uterine carcinosarcoma.
